# Effect of a light-darkness cycle on the body weight gain of preterm infants admitted to the neonatal intensive care unit

**DOI:** 10.1038/s41598-022-22533-1

**Published:** 2022-10-20

**Authors:** Manuel Sánchez-Sánchez, Teodoro L. García, Donají Heredia, Isaac Reséndiz, Lorena Cruz, Jacqueline Santiago, Adelina Rojas-Granados, Laura Ubaldo-Reyes, Laura Pérez-Campos-Mayoral, Eduardo Pérez-Campos, Gervacio S. Vásquez, Juan M. Moguel, Romeo Zarate, Oscar García, Luisa Sánchez, Fernando Torres, Alberto Paz, Jesús Elizarraras-Rivas, María T. Hernández-Huerta, Manuel Angeles-Castellanos

**Affiliations:** 1 Pediatric Division of the General Hospital “Dr. Aurelio Valdivieso”, Oaxaca City, Mexico; 2 Division de Posgrado, Facultad de Odontología, Universidad Autónoma Benito Juárez de Oaxaca, Oaxaca City, Mexico; 3 Faculty of Medicine and Surgery, Universidad Autónoma Benito Juárez de Oaxaca, Oaxaca City, Mexico; 4 Neonatology Department of the Superior Specialty Medical Unit, Hospital of Gynecology and Obstetrics N° 3. National Medical Center “La Raza”, Mexico City, Mexico; 5 Pediatric Department and Family Medicine, General Hospital of Zone No 1 “Dr. Demetrio Mayoral Pardo” and Health Research Coordination Mexican Institute of Social Security, Oaxaca City, Mexico; 6 Departamento de Anatomía, Faculty of Medicine, Universidad Nacional Autónoma de México, Edificio B 4º Piso, 04510 Mexico City, DF Mexico; 7 Tecnológico Nacional de México, Campus Oaxaca, Oaxaca City, Mexico; 8 Hospitales “Cruz Azul”, Lagunas, Oaxaca, Mexico

**Keywords:** Preterm birth, Paediatrics, Health care

## Abstract

The Continuous bright light conditions to which premature infants are subjected while hospitalized in Neonatal Intensive Care Units (NICU) can have deleterious effects in terms of growth and development. This study evaluates the benefits of a light/darkness cycle (LDC) in weight and early hospital discharge from the NICU. Subjects were recruited from three participating institutions in Mexico. Eligible patients (n = 294) were premature infants who were hospitalized in the low-risk and high-risk neonatal units classified as stable. The subjects randomized to the experimental group (n = 150) were allocated to LDC conditions are as follows: light from 07:00 to 19:00 and darkness (25 lx) from 19:00 to 07:00. The control group (n = 144) was kept under normal room light conditions (CBL) 24 h a day. Main outcome was weight gain and the effect of reducing the intensity of nocturnal light in development of premature infants. Infants to the LDC gained weight earlier, compared with those randomized to CBL, and had a significant reduction in length of hospital stay. These results highlight those premature infants subjected to a LDC exhibit improvements in physiological development, favoring earlier weight gain and consequently a decrease in hospital stays. ClinicalTrials.gov; 02/09/2020 ID: NCT05230706.

## Introduction

Worldwide, Neonatal Intensive Care Units (NICU) are often maintained under 24-h constant bright light (CBL) conditions, without significant variations in light intensity^1^. As such, infants under these conditions are exposed to chaotic temporal signals, as opposed to predictable temporal signals, without cyclic variations in terms of sounds and light. Interestingly, the level of light to which premature infants are exposed represents an accessible environmental variable that can be easily controlled^2^. Some strategies have been implemented in specific settings, but a systematic control of light conditions in the NICU is currently not in use. Premature infants are generally maintained under CBL conditions for prolonged intervals, thus hampering their growth and development^3–6^. CBL conditions can generate harmful effects to newborn eyes, and experimental studies have shown that newborn rats exposed to a CBL environment from the first day of birth develop damage in the cellular organization of the retina^7^. Previous studies have shown that a 12:12 h light/darkness cycle (LDC) can promote an increase in infant sleep time, decrease feeding time and improve weight gain, compared with infants maintained in constant light conditions^8,9^. Further, evidence shows that infants exposed to a LDC since birth acquire circadian rhythmicity of activity/rest patterns as early as week 34 of life^10^.This study presents the results from a multicentric trial which sought to determine the benefits of implementing a LDC in the NICU of three different hospitals in Mexico. The primary endpoint was weight gain; secondary endpoints included early hospital discharge, daily rhythm pattern of salivary melatonin levels and the effect of reducing the intensity of nocturnal light in development of premature infants.

## Results

A total of 294 premature infants with a mean gestational age of 32.47 ± 0.13, and a range in gestational age from 28 to 36.5 weeks of gestation from three different hospital centers in Mexico were included in this study from 2016 to 2020. General characteristics among included infants are summarized in Table 1. There were no significant differences among study groups in terms of gestational age, weight at birth, weight at discharge or daily vital signs.

**Table 1:** Characteristics of infants included in each study arm.

Variable	LL group (n = 144)	LD group (n = 150)	*P*-value
Gestational age (weeks)	32.5 ± 0.1	32.4 ± 0.2	0.633
Birth weight	1629.79 ± 31.2	1655.9 ± 26.2	0.729
Daily average heart rate	150.9 ± 0.46	149.9 ± 0.73	0.230
Daily average respiratory rate	55.7 ± 0.16	55.9 ± 0.22	0.406
Daily average temperature	37.05 ± 0.01	37.02 ± 0.02	0.311
Age at hospital discharge (week)	37.3 ± 0.2	35.8 ± 0.2	**0.0001**
Weight at hospital discharge	2168.9 ± 28.1	2242.08 ± 28.8	0.172

Significant values are in [bold].

### Feeding

**Figure 1:**
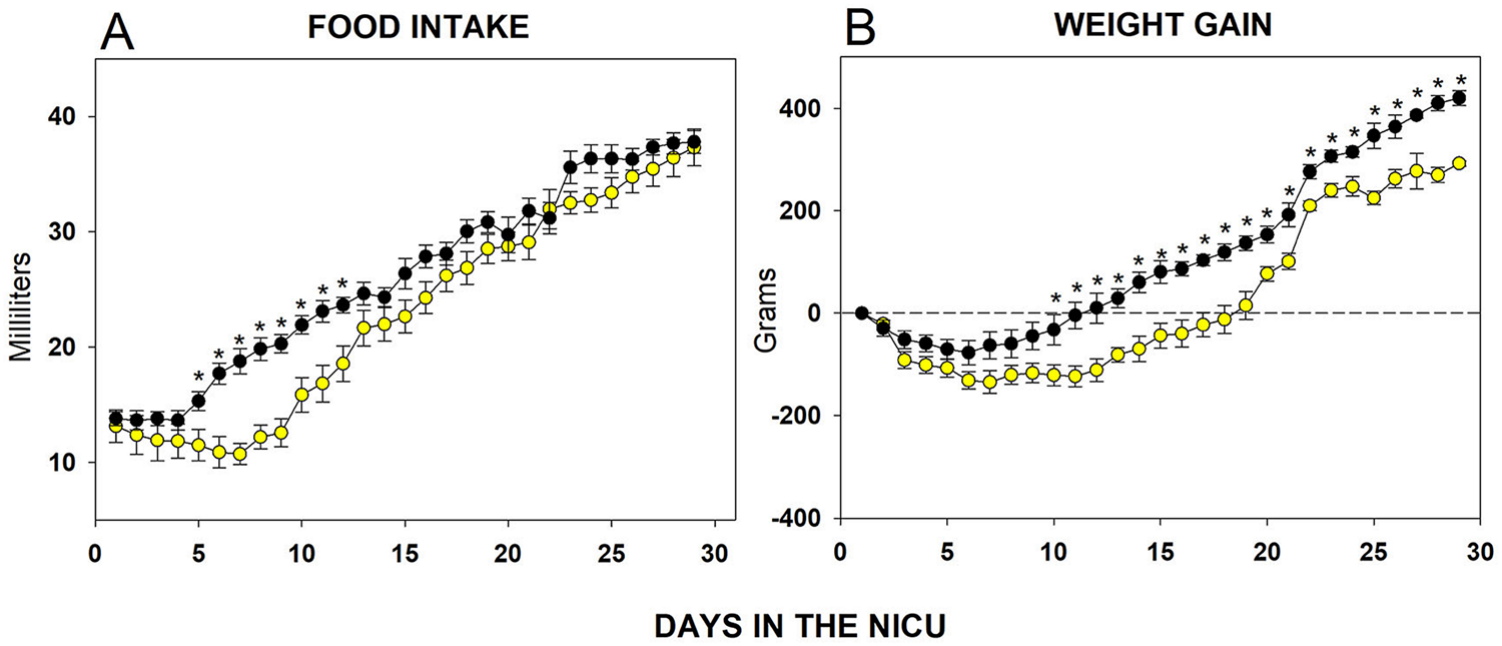
(**A**) Daily milk consumption in both study groups. Infants allocated to the experimental study group tolerated more milliliters in the initial days post-birth compared with subjects in the control group. The difference between daily milk consumption between study groups was statistically significant (*p < 0·001). (**B**) Body weight gain among infants included in each study group, there is a numerical difference since the first 7 days, but this difference is statistically significant 10 days after birth (*p < 0·001). White circles (open circle) represent the control group (CBL), while black circles (filled circle) represent the experimental group (LDC).

All infants included in the study were fed every 3 h, and received the total dose calculated for their body weight, distributed in 8 feedings throughout 24 h. During the initial 10 days of NICU hospitalization, some of the infants randomized to the CBL group presented alimentary intolerance symptoms (vomiting, diarrhea, gastric distension) as well as low milk consumption as per calculated daily dose. Nonetheless, a full recovery in feeding was observed by day 13. Infants allocated to the experimental LDC group had adequate tolerance to breast milk and had a higher milk consumption compared to infants allocated to the control group (Fig. 1A). A statistically significant difference was observed among infants included in each study group (F (1.628) = 1119; *p* < 0.0001), as well as throughout the study period (F (28.628) = 48.93; *p* < 0.0001), and in the interaction between study group and time (F28.628) = 1.96; *p* < 0.002).

### Body weight gain

Infants in both study groups had a slight decrease in body weight two days following birth (21–123 g). Thereafter, infants randomized to the CBL group had a decrease in body weight during the initial stay in the NICU, body weight then began to increase by day 12 and had recovered by day 17 of their NICU stay. Infants subjected to LDC had an increase in body weight by day seven and recovered their birth weight by day 11 of their NICU stay (Fig. 1B). The difference in body weight gain by day 21 was 128 g, favoring infants in the experimental group. The differences in terms of increase in body weight were statistically significant between study groups (F (1.897) = 100.60; p < 0.0001), over time (F (28.897) = 40.20; p < 0.0001), but not for the interaction between study group and time (F (28.897) = 1.4; p > 0.0511).

### Salivary melatonin

**Figure 2:**
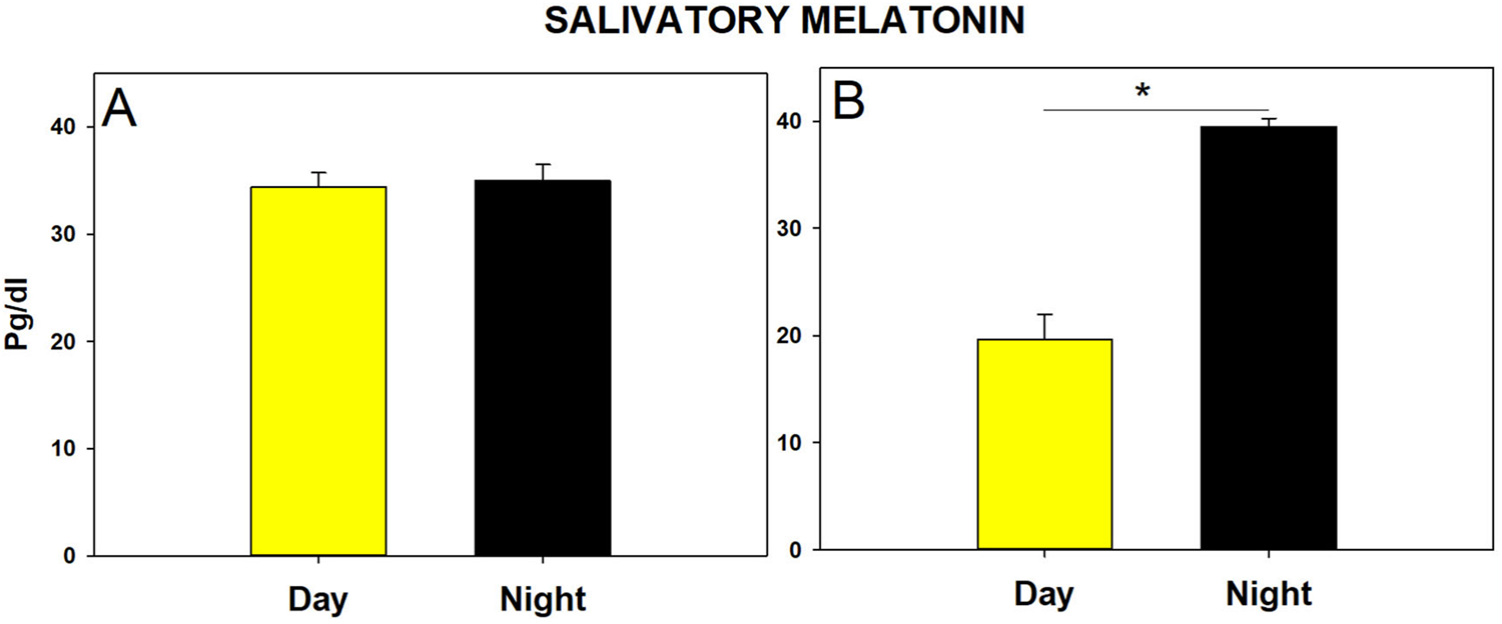
Average salivary melatonin levels; (**A**) For subjects allocated to the CBL group, there is no difference in average melatonin during light and darkness phases. (**B**) Subjects allocated to the LDC had a statistically significant difference in terms of average melatonin during day and night sample acquisition, this difference was identified since day 6 post-birth (*p* < 0·0001).

Infants allocated to the CBL group had similar melatonin levels, without significant differences between samples collected during day and night (F (1.78) = 0.625; *p* = 0.774; (Fig. 2A). The values remained constant for the initial 17 days in the NICU. In contrast, infants randomized to the LDC group had a significant difference in terms of diurnal and nocturnal melatonin levels (Fig. 2B), infants in this study group had lower levels of melatonin during the light conditions phase as early as day six, and higher levels of melatonin during the darkness conditions phase, which resulted in a daily rhythm of melatonin concentration (F (1.82) = 215.24; p < 0.00001). Melatonin levels also displayed statistically significant differences throughout time (F (16.82) = 4.16; p < 0.00001) and when assessing the interaction of study group and time (F (16.82) = 4.566; p < 0.00001) (Supplementary Fig. S1).

### Hospital discharge

**Figure 3:**
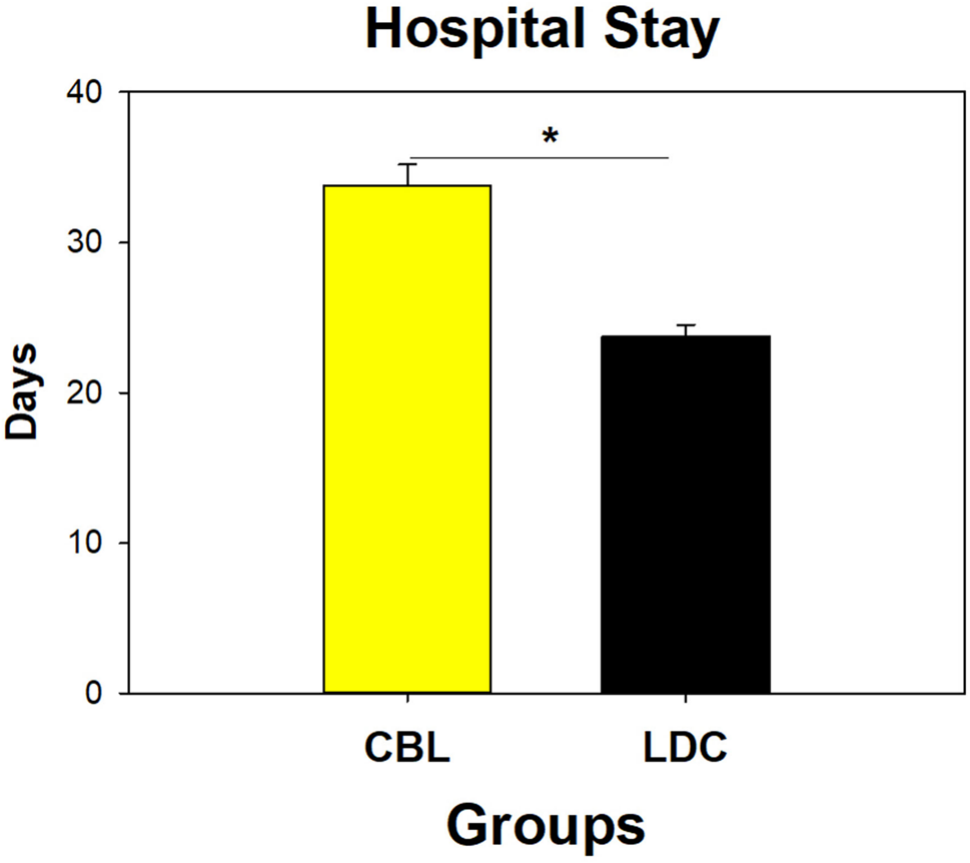
Average length of hospital stays (NICU) among subjects randomized to the control (CBL) and experimental (LDC) study groups. Patients allocated to the control group had a statistically significant prolonged hospital stay (*p < 0·001).

Among subjects included in the CBL group, the first discharge occurred on day 14 post-birth, while the last discharge occurred on day 98. Counter to this, for subjects included in the LDC group, the first hospital discharge occurred on day ten post-birth, while the last discharge occurred on day 78 (Table 1 and Fig. 3). The average length of NICU stay in the control group was 33.77 ± 1.3 days, while for the experimental group the average stay was 23 ± 0.7 days. This difference in average hospital stay was statistically significant (F (1.292) = 36.65; p < 0.0001). In the three centers we find the increase in weight, tolerance to food and early discharge. (Supplementary Table S1).

## Discussion

This multicentric study recruited subjects from three different hospital settings and implemented a LDC strategy immediately after birth to evaluate the effect of light exposure in weight gain of preterm infants. The strategy was simple and included the use of a cephalic helmet covered using a surgical cloth for 12 h a day, but through this simple manipulation we observed several beneficial events in terms of the maturity and growth of premature infants, including a better feeding tolerance and an increased and earlier onset body weight gain. These improvements further reflected in length of hospital stay, with infants randomized to the experimental group having a statistically significant improvement in this important parameter. At birth, both the subjects allocated to the control and those allocated to the experimental group had similar weights and gestational age, therefore it is very likely that the results observed in the outcomes evaluated during this study stem from the light-darkness intervention.

There is still controversy surrounding the use of LDC interventions at this early age, in terms of whether they are necessary or even beneficial towards development^11^. Nonetheless, the deleterious effects of a CBL exposure on premature infant retinas has been very well established^12^. Animal models for instance have identified damage in the cellular organization of the retina in newborn rats exposed to a constant light environment after birth^7^. Additionally, other experimental models have established a loss of circadian rhythm derived from CBL exposure^13^.

Some authors have suggested an individualized neonatal attention program, suggesting that since the *in-utero* environment is dark, infants can develop better in a constant darkness environment compared with CBL exposure^14^. Nonetheless, in utero, infants are exposed to their mothers circadian signals, which are in synch with light/darkness cycles^15^. This would suggest that the implementation of LDC immediately following birth could prove beneficial for newborns. Despite the preclinical data, to date information in this regard stemming from well-designed randomized is lacking, thus discouraging the systematic implementation of LDC as routine interventions in the NICU areas. It is important to highlight that the use of CBL in most hospital services lacks a solid scientific base^1^. In this study we identified that light intensity fluctuates from 145 to 275 lx throughout the 24 h day, and the effect of high-intensity light exposure for premature infants has been well established, showing this can lead to stress signs and physiological instability^16,17^, affecting functions involved in the visual, ocular and retinal systems of premature infants^18,19^ As such, it would be preferable to control light conditions of the NICU in order to favor the environmental adaptation of newborn infants. The positive results from our study likely stem from the light/darkness cycle, as well as the fact that the infants used a cephalic helmet. This intervention does not come in contact with the infant, as opposed to ocular protection devices which press against the face of the infant, triggering stress and increased locomotor activity. Some previous reports had sought to evaluate the effect of a LDC in a pediatric setting, for instance a study in 2003 was designed using covering blankets for the incubators, which were placed from 19:00 to 7:00 h, and removed from 7:00 to 19:00 h to achieve an LDC. Results from this trial identified a non-significant increase in body weight among subjects in both study arms^20^. Another study reported similar results, highlighting a gain in body weight reported by 14 days of hospital stay, without any significant differences reported between study arms^4^. In this last study, infants were kept either under constant dim light or constant darkness conditions, without alternating between light and darkness conditions.

Our results closely resemble a previous pilot study published in 2014 by our group. In this previous trial we included 19 infants per study group and observed an average reduction in hospital stay of 20.5 days^9^. Other studies have also reported improved outcomes among infants exposed to LDC. Mann et al*.* previously reported a faster onset weight gain and increased sleep episodes during 24 h in infants exposed to LDC, compared with those in a control group subjected to CBL^8^. This effect in terms of improved outcomes also highlights a shorter hospital stay and improved growth, especially when LDC exposure is achieved before 36 weeks of age^5^. Other benefits observed from this intervention also include a decrease in the number of days requiring supplementary oxygen for newborn infants^21^, as well as achieving earlier tolerance to feeding, which in turn generates a faster body weight gain^9^^.^

Previous studies have also identified that preterm infants have a delayed development of circadian rhythms compared with full-term newborns^22,23^, and the effect of LDC in terms of the temporal organization in general activities for newborns previous to discharge have also been evaluated^10^. An important consequence stemming from a LDC in the NICU is the increase in sleeping episodes; due to the anabolic effect of sleeping, it is likely that this could promote growth, as evidenced by increased body weight. There is evidence that the endogenous sleeping/waking pattern circadian rhythm is spontaneously developed by human infants, and that a LDC accelerates and synchronizes its appearance^24^.

Full term newborns exhibit ultradian rhythms of resting activity, sleeping through day and night for a total of 16–17 h of sleep. At the age of 3–6 postnatal weeks, the time for sleeping decreases to 14–15 h and a normal circadian pattern emerges. This process is delayed in premature infants^25^. As a result, the previous information combined with the data from this study would suggest that the sleeping-waking patter could improve due to the LDC intervention.

The ontogeny of melatonin production in infants has been studied through the assessment of 6-sulfatoximelatonin, the urinary metabolite of the hormone. In full-term infants, the circadian rhythm of melatonin is observed after eight weeks of age, while this is delayed in preterm infants, who display a rhythmic melatonin production after 12 weeks of age^26,27^. This value, however, was obtained from premature infants exposed to CBL with a luminous intensity ranging from 400 to 700 lx at the eye level of the infants. This level of light has been previously described to inhibit melatonin production in adults^28^. In this present study, infants exposed to CBL displayed a constant level of melatonin, which did not decrease during nighttime. Such observations have been also supported by a previous study in which high levels of melatonin and altered circadian rhythms were reported in infants admitted to the ICU exposed to CBL conditions^29^. In contrast, infants exposed to a LDC showed a rhythmic level of salivary melatonin, and it is likely that the alternating light conditions could have induced this rhythmic production. This finding would indicate that a well-established lighting alternate could be beneficial for the physiological maturation of the newborn, including the establishment of a rhythmic pattern in melatonin production.

This manipulation of the light/dark cycle generates direct benefits for the infants, as a reduction in hospital stay can reduce the risk of acquiring nosocomial disease as well as favor the integration of the infant to the family nucleus. Additionally, these improvements can reduce the economic expenses associated, either directly or indirectly, with an increased hospital stay. Nonetheless, most perinatology services where pre-term infants receive care have CBL conditions, without alternating between light/darkness cycles, many times due to the vigilance requirements for newborn settings, which make it difficult to establish lighting patterns in the NICU.

The data from this study should be interpreted considering its limitations, which include the lack of a progressive standardized feeding model^30^, as well as noise control in the NICU^31^ and differences in care protocols between hospitals. However, strengths include a prospective design, hospital and at-home follow-up and the inherent strengths of a multi-center design which include a larger number of recruited participants in shorter timespans, different geographic locations and the ability to compare the results between different treatment centers.

## Conclusions

Pre-term born infants who are exposed immediately to a LDC show improved physiological development, favoring body weight gain in a timely manner and a decrease in length of hospital stay. Finally, results from this study warrant further research in this field, to generate more robust data to support the role of chronobiology in perinatal medical attention.

## Methods

### Study design and participants

This prospective, open-label, randomized multicentric clinical trial, recruited patients in three different public-setting hospitals in Mexico between 2016 and 2020. Eligible patients were premature infants (gestational age < 37 weeks) who were hospitalized in the low risk and high-risk neonatal units of participating institution, with a non-severe diagnosis for hospitalization, without concomitant illness, and classified as stable. Exclusion criteria included infants hospitalized with severe illness (including any infectious, respiratory, metabolic or surgical disease which put the life of the newborn at risk and required dynamic management of solutions, medications, endotracheal intubation, surgical resolution or frequent manipulation), congenital malformations, or important neurological diseases, while elimination criteria included infants initially classified as having a non-severe illness who progress to severe illness, infants who received intensive treatment for over a week due to medical complications (i.e., bacterial infections), as well as infants whose parents requested withdrawal from participating in the study. The parents or legal guardians of all included patients provided written informed consent to participate in this study and publication of images. Participating institutions included Centro Médico Nacional "La Raza", Mexico City; Hospital General de Zona No.1 "Dr. Demetrio Mayoral Pardo" Oaxaca City, Oaxaca, Mexico (both hospitals from the public healthcare system “Mexican Institute for Social Security IMSS”), and General Hospital “Dr. Aurelio Valdivieso”, Oaxaca City, Oaxaca, Mexico (from the public healthcare system of the Health Secretariat [SSA]). The study was approved by the scientific and bioethical committees of each participating institution (CMNR-No. 3504-35-2014, HGZ 1 IMSS Oaxaca R-2001-2015-8, SSO-R. 023/2018 y UNAM-094/2014). The study was performed in accord with the declaration of Helsinky^33^ and the principles of good clinical practice. This study followed the Consolidated Standards of Reporting Trials (CONSORT) reporting guidelines. (ClinicalTrials.gov; 02/09/2020 ID: NCT05230706).

### Sample size

The sample size was calculated for a two-sample comparison, given an 80% sensitivity and a 60% specificity and with a confidence interval of 90%. A total of 150 patients per group was estimated, for a total sample size of 300 subjects. After randomization, were excluded (n = 6), from the group CBL, due to the start of the COVID-19 pandemic, the hospitals were closed, and we were unable to continue monitoring these children.

### Randomization and masking

Eligible patients were randomly assigned in a 1:1 ratio using sequential randomization to the experimental (LDC) or control (CBL) arm of the study in all participating center. The first recruited infant was assigned to the CBL control group, while the second was assigned to the LDC experimental group, and so on. The method was simple randomization; the study was not masked to the primary care physician or the parents of included patients, however, the investigators who performed the analyses were blinded to the allocation.

### Outcomes

The primary outcome was weight gain, which was determined by daily weighting (08:00–09:00 h of every morning) using a pediatric precision scale (Guian); the result was subtracted from the previous day registered weight to obtain the total daily weight gain. Results were further organized to determine the body weight gain during the initial 29 days of hospitalization. Secondary outcomes included early hospital discharge and daily rhythm pattern of salivary melatonin levels.

### Procedures

**Figure 4:**
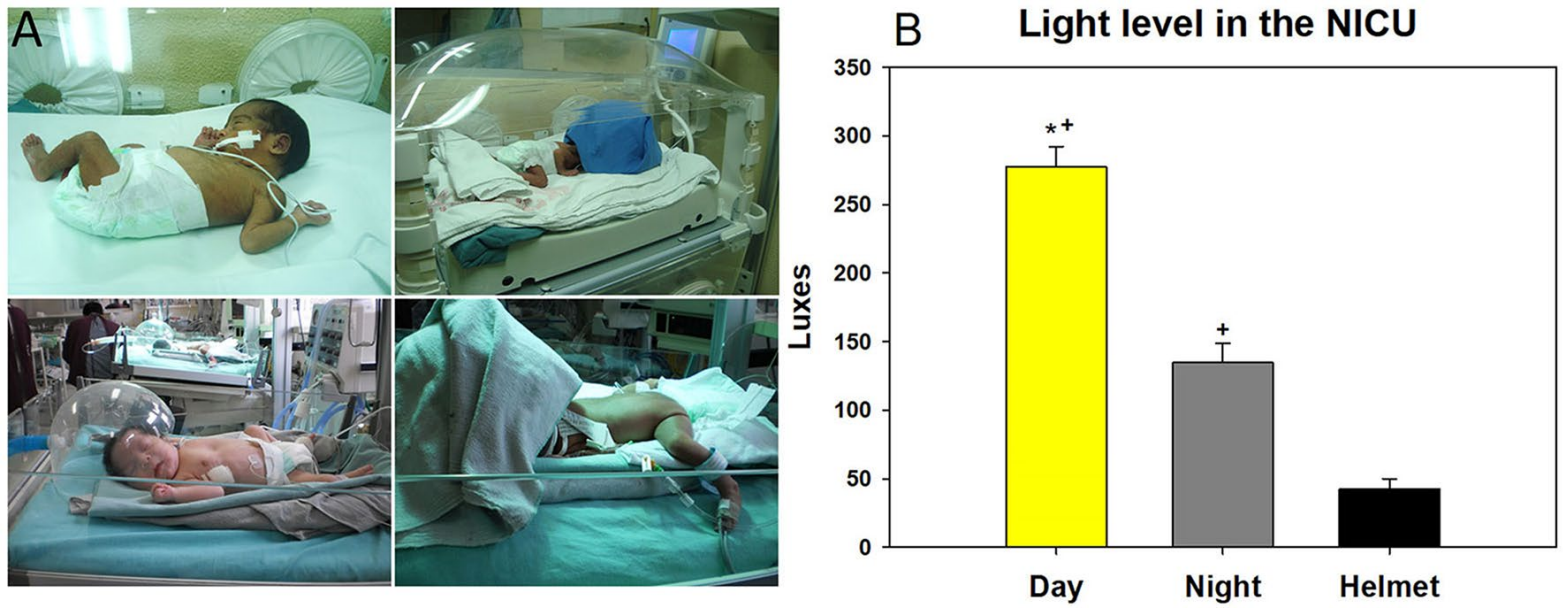
(**A**) Representative images of infants randomized to CBL (left panels) and LDC (right panels). (**B**) Illumination level (measured in Lux mean ± SD) during different conditions in the NICU, including daytime, nighttime, and when using the cephalic helmet for infants randomized to the LDC study group (*p-value < 0·002; + *p*-value < 0·001 when comparing with the illumination level while wearing the cephalic helmet); (the authors have the consent of the parents of the patients to publish the photographs).

All study procedures were performed following a predefined study protocol and a uniform sample processing plan. Data was registered using a unified registry form in all participating sites, and all analyses were centrally performed at the main investigation site. All included patients provided information regarding family and perinatal history. Each NICU from the participating institutions was evaluated to measure the illumination level using a luxmeter (HER-410-Steren), in 4 specific areas during 6 different times of day to ascertain homogeneous lighting conditions throughout the centers. Subjects randomized to the experimental group were allocated to alternating light/darkness conditions as follows: from 07:00 to 19:00 h the subjects were kept under normal room light conditions; from 19:00 to 07:00 of the following day the conditions were modified by placing the patient under an acrylic cephalic helmet (length: 27 cm; width: 27 cm; height: 17.5 cm; opening: 17 × 12 cm). The helmet was covered with surgical cloth (green or blue) folded to 50 × 60 cm rectangles and was measure the illumination level under the cover, leaving the frontal part open to maintain an adequate air flow. The procedures performed had been assessed for safety and feasibility in a previous study^9^. This intervention exposed infants in the experimental group to light at 25 lx for 12 h overnight (Fig. 4A, right), while during daytime the cloth was removed for study subjects to be exposed to regular room lighting (Fig. 4A left). The control group was kept under normal room light conditions (CBL) 24 h a day (level of illumination was 275.82 ± 14 lx during the day and 145.28 ± 14 lx at night, Fig. 4B). It is important to mention that in the participating NICUs there is no standardized practice to protect the newborn from constant lighting. Also, the NICU space in all centers is exposed to ceiling lights alone, without windows or shutters to be manipulated by the staff. All included subjects were under 24-h surveillance for the period spanning the length of their hospital stay by the NICU personnel at each participating institution. Infants were kept in individual incubators regulated to maintain a body temperature ranging from 36.5 to 37 °C. Both study groups were offered feeding and vital signs were recorded every three hours (cardiac and respiratory rate, temperature). Every infant underwent an 8–12 h fast after birth for intestinal resting, thereafter enteral feeding began at a dose of 12.5 ml/kg of breast milk, which was increased every 12 h until reaching a maximum of 200 ml/kg/day; the total caloric content of foods was standardized between different hospitals at 40–60 kcal/kg/day. Every included infant was orally fed, in case of abdominal distension or absence of peristalsis they underwent a 24-h maximum fast. Once the infants reached 1600 g body weight, breast feeding was indicated to prepare for discharge. Among 20 randomly selected infants to comparable groups (10 randomized to BCL and 10 randomized to LDC) saliva samples were collected two times a day (08:00, 23:00 h) every day during the first 20 days of NICU stay to determine biomarkers. The salivary concentration of melatonin was determined using a standardized ELISA kit, following instructions by the manufacturer (IBL International GmbH Flughafenstr. Hamburg, Germany). Other procedures performed included capillary blood glucose brad, administration of medication as prescribed by their primary care physician, and sample acquisition for laboratory testing pertaining each condition presented by infants included in this trial. All included patients were monitored four weeks post hospital discharge to evaluate development and growth at home through questionnaires which were filled out by the parents and included data regarding feeding time, sleeping hours and signs of dyspepsia. Healthy premature infants were considered for discharge when they presented an adequate growth curve and met established growth criteria^33^, including the ability to maintain a stable body temperature in an open crib, ability to perform all feedings without respiratory distress, and a constant weight gain > 2000 g.

### Statistical analysis

Continuous variables were summarized as arithmetic means with standard deviations or medians and ranges according to the data distribution assessed with the Kolmogorov–Smirnov test. For descriptive purposes, categorical variables were summarized as frequencies and percentages. Data pertaining to body weight gain, feeding and melatonin concentration were classified per study group and evaluated as repeated measures according to post-natal days. Comparisons among study groups were performed using a two-way ANOVA, followed by a TUKEY post-test analysis. A p-value < 0.05 was considered statistically significant. The initial body weight, gestational week, and length of hospital stay were compared using a one-way ANOVA, a p-value < 0.05 was considered statistically significant. All analyses were performed using the STATISTICA program (Version 10; Stat Soft, Inc. 1993), while figures were constructed using the program Sigma Plot (Version14; Systat Software, San Jose, CA). www.systatsoftware.com

## Supplementary Information

Supplementary Information 1.

Supplementary Information 2.

## Data Availability

The full trial protocol, and some data will be available at: https://anatomia.facmed.unam.mx/index.php/jefa-de-seccion-academica-investigacion and requests for materials should be addressed to M.A.C. TRN: Trainee Research Network of Clinical and Experimental Chronobiology.
