# Supplementary figures and images for: Effect of a light-darkness cycle on the body weight gain of preterm infants admitted to the neonatal intensive care unit
Source: Sci Rep. 2022 Oct 20;12:17569. doi: 10.1038/s41598-022-22533-1 (PMC9584226; doi:10.1038/s41598-022-22533-1)

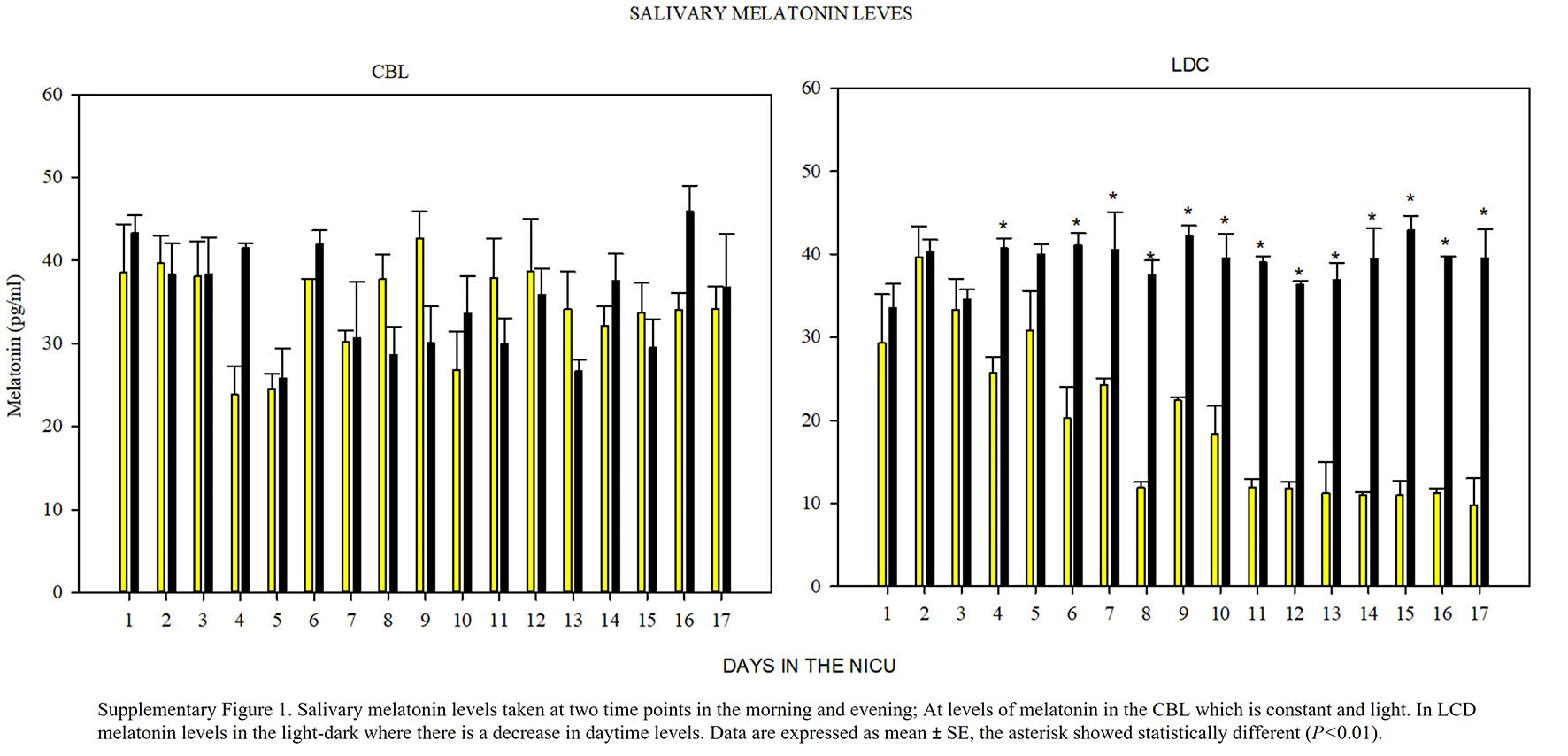

Supplement: Supplementary file 1 — Supplementary Information 1. [file 41598_2022_22533_MOESM1_ESM.jpg]
