# Supplementary material for: Effect of a light-darkness cycle on the body weight gain of preterm infants admitted to the neonatal intensive care unit
Source: Sci Rep. 2022 Oct 20;12:17569. doi: 10.1038/s41598-022-22533-1 (PMC9584226; doi:10.1038/s41598-022-22533-1)
Supplement: Supplementary file 2 — Supplementary Information 2. [file 41598_2022_22533_MOESM2_ESM.docx]

Supplementary Table 1

Clinical course according to each hospital center

| HOSPITAL CENTERS | **Gestational age (wk)** | | **Birth weight,**  **(g)** | | **Discharge weight, (g)** | | **Length of stay**  **(day)** | | **Difference in time of hospital stay (days)** |
| --- | --- | --- | --- | --- | --- | --- | --- | --- | --- |
|  | CBL | LDC | CBL | LDC | CBL | LDC | CBL | LDC |  |
| CMNR | 31.7±0.2 | 31.5±0.2 | 1698.5±29 | 1725.9±31 | 2112.4±25 | 2193.1±32 | 30.7±0.7 | **19.2±0.4*** | **11.5** |
| GHDAV | 33.1±0.3 | 32.8±0.4 | 1629.6±74 | 1617±59 | 2259.5±71 | 2189.9±50 | 34.8±3.5 | **26.1±1.9*** | **8.7** |
| HGZ1 | 33.3±0.3 | 33.4±0.4 | 1510.6±71 | 1574±77.2 | 2171.6±60 | 2399.2±75 | 38.8±3.5 | **29.2±2.3*** | **9.6** |

CBL: Constant Bright Light

LDC: Light Dark Cycle

CMNR: Centro Médico Nacional "La Raza"

HGDAV: General Hospital “Dr. Aurelio Valdivieso

HGZ1: Hospital General de Zona No.1

(*): P<0.005
